# Physical-Layer Security Improvement with Reconfigurable Intelligent Surfaces for 6G Wireless Communication Systems

**DOI:** 10.3390/s21041439

**Published:** 2021-02-19

**Authors:** Janghyuk Youn, Woong Son, Bang Chul Jung

**Affiliations:** Department of Electronics Engineering, Chungnam National University, Daejeon 34134, Korea; jhyoon@o.cnu.ac.kr (J.Y.); woongson@cnu.ac.kr (W.S.)

**Keywords:** physical-layer security, reconfigurable intelligent surface, intelligent reflecting surface, secure communication, passive eavesdropper, 6G wireless communication system, tera-hertz spectrum

## Abstract

Recently, reconfigurable intelligent surfaces (RISs) have received much interest from both academia and industry due to their flexibility and cost-effectiveness in adjusting the phase and amplitude of wireless signals with low-cost passive reflecting elements. In particular, many RIS-aided techniques have been proposed to improve both data rate and energy efficiency for 6G wireless communication systems. In this paper, we propose a novel RIS-based channel randomization (RCR) technique for improving physical-layer security (PLS) for a time-division duplex (TDD) downlink cellular wire-tap network which consists of a single base station (BS) with multiple antennas, multiple legitimate pieces of user equipment (UE), multiple eavesdroppers (EVEs), and multiple RISs. We assume that only a line-of-sight (LOS) channel exists among the BS, the RISs, and the UE due to propagation characteristics of tera-hertz (THz) spectrum bands that may be used in 6G wireless communication systems. In the proposed technique, each RIS first pseudo-randomly generates multiple reflection matrices and utilizes them for both pilot signal duration (PSD) in uplink and data transmission duration (DTD) in downlink. Then, the BS estimates wireless channels of UE with reflection matrices of all RISs and selects the UE that has the best secrecy rate for each reflection matrix generated. It is shown herein that the proposed technique outperforms the conventional techniques in terms of achievable secrecy rates.

## 1. Introduction

Reconfigurable intelligent surfaces (RISs) or intelligent reflecting surfaces (IRSs) have been proposed to achieve high spectral and energy efficiency for future 6G wireless communication systems [1,2,3,4]. An RIS consists of a large number of passive elements, each of which can independently change the received signal and reflect the altered signal. For the passive elements that can reflect, conventional reflect-arrays, liquid crystal surfaces, or software-defined meta-surfaces can be applied [5]. In particular, millimeter-wave (mmWave) and tera-hertz (THz) communication are known to be well-compatible with the RISs for line-of-sight (LOS) environments, since mmWave and THz communications suffer from significant path-loss in general. There exist several studies related to the RIS in LOS environments for mmWave and THz communications. For example, an RIS-aided joint optimization technique of the transmit beam forming at the access point (AP) and passive phase-shift at the RIS was investigated to enhance the energy efficiency at the AP in RIS-aided multi-user multiple-input single-output (MISO) communication systems [6]. An RIS-based phase-shift technique for effective ranking with a singular value of the RIS-augmented channel maximization was investigated in RIS-aided multi-user single-input multiple-output (SIMO) communication systems [7]. In addition, RIS was applied to a mobile edge computing system for maximizing the sum of computational bits, since the benefits of RISs can be exploited to enhance computing performance [8]. Moreover, it was shown that the RISs can improve the ranks of LOS MIMO communication systems [9].

Meanwhile, physical-layer security (PLS) has received considerable attention from both academia and industry [10]. Many studies have been performed to enhance PLS for future wireless networks, such as massive MIMO, non-orthogonal multiple access (NOMA), Internet of Things (IoT) networks, and mmWave and THz communications [11,12,13]. The eavesdropping attack scenario in which a malicious device attempts to overhear a private message between legitimate devices is constantly being considered as an important security issue in the literature [14]. Eavesdropping scenarios with the PLS can be classified into three scenarios: passive, active, and potential eavesdropping scenarios [15,16,17,18,19,20,21,22,23]. A passive eavesdropper (EVE) always attempts to overhear the private message and does not take any other proactive actions [15,16,17,18,19]. By contrast, an active EVE not only attempts to overhear the private message, but also induces some malfunction, such as artificial noise, a jamming attack, pilot contamination, or fake information feedback [16,17]. The concept of the potential EVE was introduced in [20,21,22,23]. The term potential is used in the sense that the EVEs may operate as legitimate devices in some instances. For example, in multi-user uplink cellular networks, all unscheduled legitimate users in a certain cell are defined as potential EVEs in [21], and some of the unscheduled users in a certain cell are defined as potential EVEs in [22]. Furthermore, in multi-user downlink cellular networks, potential EVEs can attempt to overhear legitimate communications of other cellphones or participate in their own legitimate communications [20,23].

Recently, it was shown that the performance of PLS can be improved by adding RISs to wireless communication systems [24,25,26,27,28,29,30,31,32]. These techniques have been proposed to improve the secrecy rate by maximizing the legitimate channel gain and minimizing the eavesdropping channel gain for private message transmission through the legitimate link. Furthermore, joint transmit beamforming and reflecting beamforming optimization with artificial noise (AN) or jamming signals have been proposed for PLS enhancement in various wireless networks [33,34,35,36,37,38,39]. These techniques aim to maximize the signal to interference-plus-noise ratio (SINR) for a legitimate user and to minimize SINR for the EVE by transmitting a signal that combines the private message and AN. There have been several studies that considered legitimate links which were reflected only on RISs due to some obstacle [29,30,33], and some giving imperfect channel state information (CSI) to EVEs [35,38]. In addition, the multiple RIS-based joint transmit beamforming and AN optimization technique considering a potential eavesdropping attack scenario for PLS enhancement was proposed in [36]. However, in the literature, most studies considered iterative heuristic or complex algorithms for optimization, which require significant signaling overheads among the BS, RISs, and user equipment (UE), and if necessary, EVEs. Thus, a practical PLS improvement technique with RISs that does not involve a significant signaling overhead is required.

In this paper, we propose a novel RIS-based channel randomization (RCR) technique for improving PLS for a time-division duplex (TDD) downlink cellular wire-tap network consisting of a single BS, multiple legitimate pieces of UE, multiple passive EVEs, and multiple RISs. Each RIS pseudo-randomly generates multiple reflection matrices and utilizes them for pilot signal duration (PSD) in uplink and data transmission duration (DTD) in downlink. The BS estimates all wireless channels to the UE, including reflected wireless channels at IRSs, and selects the UE which can be achieve the best secrecy rate for each reflection matrix generated. As a result, our main contributions can be summarized as follows:

- We propose an RCR technique that repeats some random channel by repeating a certain number of reflecting matrices of the RIS. With the RCR technique, we can design a repeating random communication channel, possibly even in a short enough time that the communication channel will not be changed.
- Based on the RCR technique, user scheduling for each random channel was performed to maximize the secrecy rate. It was shown that the proposed RCR-based scheduling technique can achieve better performance than the network without any RIS and than a random scheduling technique.

The rest of paper is composed as follows. In Section 2, the considered system model is described. Then the overall procedure of proposed RCR technique is explained in Section 3. The numerical results are shown in Section 4 and the proposed RCR technique is compared with reference techniques. Finally, the conclusion of this paper is written in Section 5.

## 2. System and Channel Model

**Figure 1:**
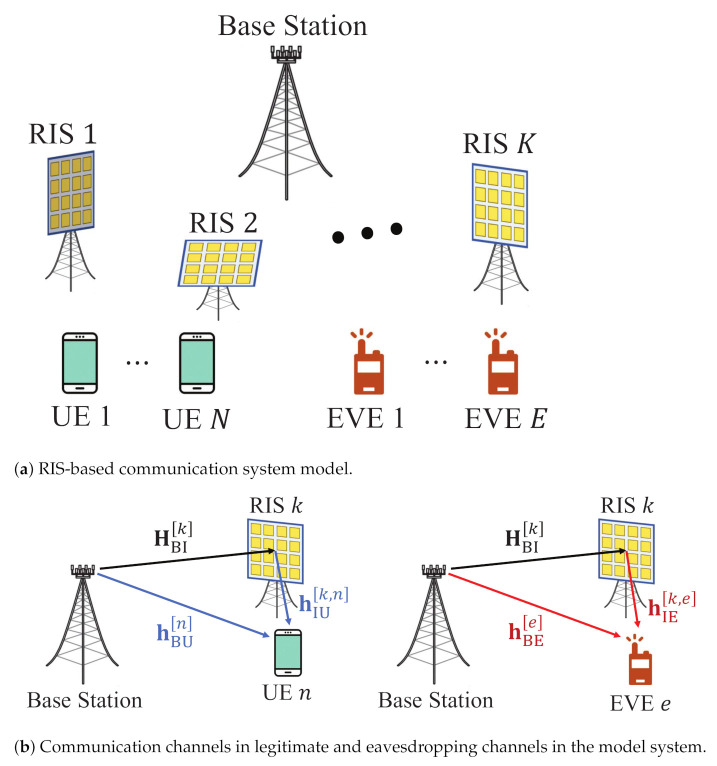
RIS-based communication model and channel model.

In Figure 1a, the considered system in this paper is described, which consists of a BS, multiple RISs (*K*), multiple pieces of UE (*N*), and multiple EVEs, *E*. Note that EVE is the potential EVE—one of the pieces of user equipment outside of the cell, which transmits a pilot signal for its own communication. However, here, EVEs can overhear the other pieces of UE’s communications and decode the private messages which are transmitted for other legitimate UE. With this assumption, the BS can acquire the CSI with EVEs by receiving the pilot signal from EVEs. Moreover, the BS is equipped with transmit antennas (*M*), RISs are equipped with multiple passive reflecting elements (*L*), and all UE and all EVEs are equipped with a single receiving antenna. For the BS and RISs, the uniform linear array (ULA) structure is adopted for transmitting antennas and passive reflecting elements, respectively.

As described in Figure 1b, the communication channels from the BS to the *k*-th RIS, from the BS to the *n*-th piece of UE, and from the BS to the *e*-th EVE are represented by $H_{\mathrm{BI}}^{\left[k\right]}$, $h_{\mathrm{BU}}^{\left[n\right]}$, and $h_{\mathrm{BE}}^{\left[e\right]}$, respectively. In addition, $h_{\mathrm{IU}}^{\left[n\right]}$ and $h_{\mathrm{IE}}^{\left[e\right]}$ respectively denote the communication channel from the RIS to the *n*-th piece of UE and from the RIS to the *e*-th EVE. We assume that all channels in the considered system are LOS channels, since an extremely high frequency band will be utilized in 6G. Hence, all communication channels are defined as follows.

(1) $$\begin{array}{c} \begin{array}{c} H_{\mathrm{BI}}^{\left[k\right]}=\sqrt{ML}a_{L}\left(\theta_{\mathrm{BI},R}^{\left[k\right]}\right)a_{M}{\left(\theta_{\mathrm{BI},T}^{\left[k\right]}\right)}^{H},\\ h_{\mathrm{BU}}^{\left[n\right]}=\sqrt{M}a_{M}{\left(\theta_{\mathrm{BU},T}^{\left[n\right]}\right)}^{H},\\ h_{\mathrm{BE}}^{\left[e\right]}=\sqrt{M}a_{M}{\left(\theta_{\mathrm{BE},T}^{\left[e\right]}\right)}^{H},\\ h_{\mathrm{IU}}^{\left[n\right]}=\sqrt{L}a_{L}{\left(\theta_{\mathrm{IU},T}^{\left[n\right]}\right)}^{H},\\ h_{\mathrm{IE}}^{\left[e\right]}=\sqrt{L}a_{L}{\left(\theta_{\mathrm{IE},T}^{\left[e\right]}\right)}^{H}, \end{array} \end{array}$$

where $a_{X}\left(\theta \right)={\left[1,e^{-j\pi \theta },\cdots ,e^{-j\pi \left(X-1\right)\theta }\right]}^{T}$ is a steering vector for a given number of antennas, *X* is the angle of arrival or angle of departure, and θ is half of the wavelength antenna spacing. Moreover, $\theta_{X,T}^{\left[x\right]}$ and $\theta_{X,R}^{\left[x\right]}$ represent angle of departure and angle of arrival for corresponding channel $H_{X}^{\left[x\right]}$ or $h_{X}^{\left[x\right]}$, respectively. In addition, we assume that all LOS channels do not change in the course of a single transmission, but change after transmission ends.

## 3. The RIS-Based Channel Randomization Technique for Secure Communication

In this section, we describe the overall procedure of the proposed RIS-based channel randomization (RCR) technique for secure communication. Conceptually, RISs in the proposed technique repeat the reflection matrix upon pilot signal transmission and data signal transmission; reflection matrices are randomly generated.

**Figure 2:**
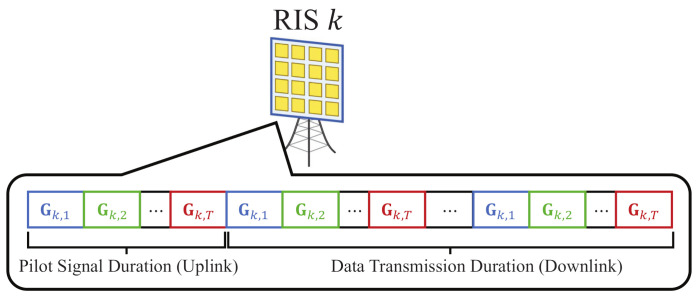
RIS reflection matrix shifting in pilot signal duration and data signal duration.

First of all, we separate the communication duration into a pilot signal duration (PSD) and data transmission duration (DTD). PSD has *T* time slots and DTD has βT time slots, where β is a natural number. Before the transmission procedure starts, each *k*-th RIS generates *T* random reflection matrices, which correspond to *T* time slots in PSD, as shown in Figure 2. Then, the reflection matrix of the *k*-th RIS that corresponds to the *t*-th time slot is denoted as $G_{k,t}$, and the set of reflection matrices for PSD is derived as follows.

(2) $$\begin{array}{c} G_{k}=\left\{G_{k,1},G_{k,2},\cdots ,G_{k,T}\right\}, \end{array}$$

where the reflection matrix $G_{k,t}$ is defined as follows

(3) $$\begin{array}{c} G_{k,t}=\left[\begin{array}{cccc} e^{j\phi_{k,t}^{\left[1\right]}} & 0 & \cdots & 0\\ 0 & e^{j\phi_{k,t}^{\left[2\right]}} & \cdots & 0\\ \vdots & \vdots & \ddots & \vdots \\ 0 & 0 & \cdots & e^{j\phi_{k,t}^{\left[L\right]}} \end{array}\right], \end{array}$$

where $\phi_{k,t}^{\left[l\right]}$ represents the random phase of the *l*-th passive reflecting element in the *k*-th RIS at the *t*-th time slot.

After the reflection matrix set is generated, all UE and EVEs transmit pilot signal to the BS during all time slots over the PSD. Then, the BS acquires all CSI between itself and UE or EVEs; thus, the CSI acquired with the *n*-th UE and the *e*-th EVE in *t*-th time slot can be derived as follows:

(4) $$\begin{array}{c} \begin{array}{c} h_{U}^{\left[n,t\right]}=h_{\mathrm{BU}}^{\left[n\right]}+\sum_{k=1}^{K}h_{\mathrm{IU}}^{\left[k,n\right]}G_{k,t}H_{\mathrm{BI}}^{\left[k\right]},\\ h_{E}^{\left[e,t\right]}=h_{\mathrm{BE}}^{\left[e\right]}+\sum_{k=1}^{K}h_{\mathrm{IE}}^{\left[k,e\right]}G_{k,t}H_{\mathrm{BI}}^{\left[k\right]}, \end{array} \end{array}$$

respectively. Note that the reflection matrices of the RIS are the only time varying parameter of the channel between the BS and the UE or EVE, since we assume there are only LOS channels between nodes. Now, based on acquired CSI, BS allocates UE to each *t*-th time slot based on the following allocation metric:

(5) $$\begin{array}{c} n_{t}=\underset{n}{\mathrm{argmax}}\frac{{\left|h_{U}^{\left[n,t\right]}\right|}^{2}}{\max_{e}\left|h_{E}^{\left[e,t\right]}\right|}, \end{array}$$

where $n_{t}$ is the UE index that is allocated to the *t*-th time slot, and this allocation metric makes the BS allocate each *n*-th UE to the specific time slot that should achieve the best secrecy rate. In addition, since the BS is equipped with *M* transmit antennas, the BS can serve the maximum amount of UE for simultaneous transmission in a single time slot. Here, we define *S* as a maximum number of UE selected for each time slot. However, since the goal of the proposed scheduling technique is secure communication, the BS also needs to manage the leakage to EVEs by beamforming. It is obvious that the BS can sufficiently manage the leakage to EVEs when the number of antennas is much larger than selected UE, i.e., S≪M. Hence, we set the maximum number of selected UE to be much smaller than *M*.

To select *S* UE, the selection metric of (5) is performed *S* times. Then, the set of users that allocated to the *t*-th time slot can be derived as follows:

(6) $$\begin{array}{c} N_{t}=\left\{n_{t}^{\left[1\right]},n_{t}^{\left[2\right]},\cdots ,n_{t}^{\left[S\right]}\right\}, \end{array}$$

where $n_{t}^{\left[s\right]}$ is *s*-th selected UE at the *t*-th time slot. As a result, the allocation process of the proposed technique can be summarized as Algorithm 1.
**Algorithm 1** Time slot allocation algorithm for user equipment (UE) scheduling.**Input:**$h_{U}^{\left[n,t\right]}$, $h_{E}^{\left[e,t\right]}\;\forall n,\forall e,\forall t$**Output:**$N_{t}\;\forall t$ **for**
t=1:T
**do**  $N=\left\{1,2,\cdots ,N\right\},\;N_{t}=\emptyset$  **for**
s=1:S
**do**   $n_{t}^{\left[s\right]}=\underset{n\in N}{\mathrm{argmax}}\frac{{\left|h_{U}^{\left[n,t\right]}\right|}^{2}}{\max_{e}{\left|h_{E}^{\left[e,t\right]}\right|}^{2}}$   $N_{t}=N_{t}\cup n_{t}^{\left[s\right]},\;N=N\backslash n_{t}^{\left[s\right]}$  **end for** **end for**


After UE scheduling is over for all time slots, the BS designs its transmit beamforming for each time slot based on the CSI of selected UE. Note that the target of the transmit beamforming design of the BS is to maximize the power received at UE while minimizing the power received at EVEs, which means maximizing the secrecy rate. Meanwhile, there is already an ideal solution for designing a beamforming matrix for the maximum secrecy rate wherein both the communication channel and the eavesdropping channel are taken into account [40]. Based on the solution of [40], the BS designs a transmitting beamforming matrix for each time slot for the communication channel of a selected UE and that of EVEs as follows:

(7) $$\begin{array}{c} B_{t}={\mathrm{Eig}}_{S}\left({\left(I_{M}+\rho H_{E,t}^{H}H_{E,t}\right)}^{-1}\left(I_{M}+\rho H_{C,t}^{H}H_{C,t}\right)\right), \end{array}$$

where $H_{C,t}$ represents a communication channel matrix consisting of *S* channel vectors of UE that are scheduled for the *t*-th time slot; i.e., $H_{C,t}={\left[h_{C}^{\left[n_{t}^{\left[1\right]},t\right]T},h_{C}^{\left[n_{t}^{\left[2\right]},t\right]T},\cdots ,h_{C}^{\left[n_{t}^{\left[S\right]},t\right]T}\right]}^{T}$. In addition, $H_{E,t}$ is an eavesdropping channel matrix consisting of channel vectors of EVEs, i.e., $H_{E,t}={\left[h_{E}^{\left[1,t\right]T},h_{E}^{\left[2,t\right]T},\cdots ,h_{E}^{\left[E,t\right]T}\right]}^{T}$. The ρ and $I_{M}$ indicate transmit signal-to-noise ratio (SNR) and an identity matrix that is M×M in size, respectively. Moreover, ${\mathrm{Eig}}_{S}\left(\cdot \right)$ denotes a function for which the output is *S* eigenvectors that correspond to the highest *S* eigenvalue.

Furthermore, for the case that multiple pieces of UE are allocated to a single time slot, the inter-user interference needs to be managed. Hence, the BS designs a zero-forcing matrix (a well-known matrix) to eliminate the inter-user interference in a single time slot as follows.

(8) $$\begin{array}{c} Z_{t}={\left(H_{C,t}B_{t}\right)}^{-1}. \end{array}$$

As a result, the final beamforming matrix for the *t*-th time slot in which only one UE is scheduled is $V_{t}=B_{t}$. By contrast, if S>1, then the final beamforming matrix for the *t*-th time slot is $V_{t}=B_{t}Z_{t}$, but it is normalized to each of its column vectors that have unit power. It is worth noting that there is no additional signaling process in the overall procedure of the proposed RCR technique except channel estimation. Since the RCR technique does not require information exchange between BS and RIS, it can be easily implemented to practical communication systems. Briefly, the transmit beamforming matrix design of BS can be described as Algorithm 2.
**Algorithm 2** Transmit beamforming design at the base station (BS) for all time slots.**Input:***S*, $h_{U}^{\left[n,t\right]}$, $h_{E}^{\left[e,t\right]},N_{t}\;\forall n,\forall e,\forall t$**Output:**$V_{t}\;\forall t$ **for**
t=1:T
**do**  $H_{C,t}={\left[h_{C}^{\left[n_{t}^{\left[1\right]},t\right]T},h_{C}^{\left[n_{t}^{\left[2\right]},t\right]T},\cdots ,h_{C}^{\left[n_{t}^{\left[S\right]},t\right]T}\right]}^{T}$  $H_{E,t}={\left[h_{E}^{\left[1,t\right]T},h_{E}^{\left[2,t\right]T},\cdots ,h_{E}^{\left[E,t\right]T}\right]}^{T}$  $B_{t}={\mathrm{Eig}}_{S}\left({\left(I_{M}+\rho H_{E,t}^{H}H_{E,t}\right)}^{-1}\left(I_{M}+\rho H_{C,t}^{H}H_{C,t}\right)\right)$   **if**
S>1
**then**   $Z_{t}={\left(H_{C,t}B_{t}\right)}^{-1}$   $V_{t}=B_{t}Z_{t}$  **else**   $V_{t}=B_{t}$  **end if**  All column vectors of $V_{t}$ are normalized to the unit norm.**end for**

After beamforming design is over, the DTD starts. Note that DTD consists of βT time slots as described in the beginning of this chapter. Moreover, in DTD, all RISs repeat the same reflection matrices β times. As a result, it is obvious that all UE and EVEs will use exactly the same communication channel in the DTD as in the PSD β. Therefore, the BS can utilize the designed beamforming matrices, $V_{t}$, in DTD, since the same channels as for PSD will be repeated in DTD. Then, the signal received at the *n*-th UE and the *e*-th EVE in the *t*-th time slot can be derived as follows.

(9) $$\begin{array}{c} \begin{array}{c} y_{n,t}=h_{U}^{\left[n,t\right]}V_{t}x_{n,t}+z_{n,t},\\ y_{e,t}=h_{E}^{\left[e,t\right]}V_{t}x_{n,t}+z_{e,t}, \end{array} \end{array}$$

respectively, where $x_{n,t}$ is the original signal of *n*-th UE at the *t*-th time slot. In addition, $z_{n,t}$ and $z_{e,t}$ are the additive Gaussian noise of *n*-th UE and *e*-th EVEs in the *t*-th time slot, which follows either a zero mean or a unit variance Gaussian distribution, i.e., $\mathrm{CN}\left(0,1\right)$, respectively.

Finally, the secrecy rate performance of the proposed RCR technique can be calculated as follows.

(10) $$\begin{array}{c} \mathrm{SR}=\frac{1}{T}\sum_{t=1}^{T}\sum_{s=1}^{S}\left[\log_{2}\left(1+\rho {\left|h_{U}^{\left[n_{t}^{\left[s\right]},t\right]}v_{t}^{\left[s\right]}\right|}^{2}\right)-\log_{2}\left(1+\rho \;\max_{e}{\left|h_{E}^{\left[e,t_{n}\right]}v_{t}^{\left[s\right]}\right|}^{2}\right)\right], \end{array}$$

where $V_{t}=\left[v_{t}^{\left[1\right]},v_{t}^{\left[2\right]},\cdots ,v_{t}^{\left[S\right]}\right]$. Here, the physical meaning of the secrecy rate is the maximum data rate that can be securely delivered to legitimate users without leakage to eavesdroppers.

## 4. Numerical Results

In this section, we validate the performance of proposed RCR technique in terms of secrecy rate. For the simulation, we used Matlab 2019a software and Monte Carlo simulation method by realizing random channel for each iteration. Moreover, we set the two reference technique which are non-RIS case and random scheduling for the comparison with proposed RCR technique. Non-RIS case is that only direct LOS channel between BS and UE or EVE is available, since there is no RIS in the network. Hence, only optimal beamforming design of BS is utilized for PLS enhancement. In addition, random scheduling is that UE are randomly scheduled at time slot. Note that also in random scheduling, BS designs optimal transmit beamforming as [40].

**Figure 3:**
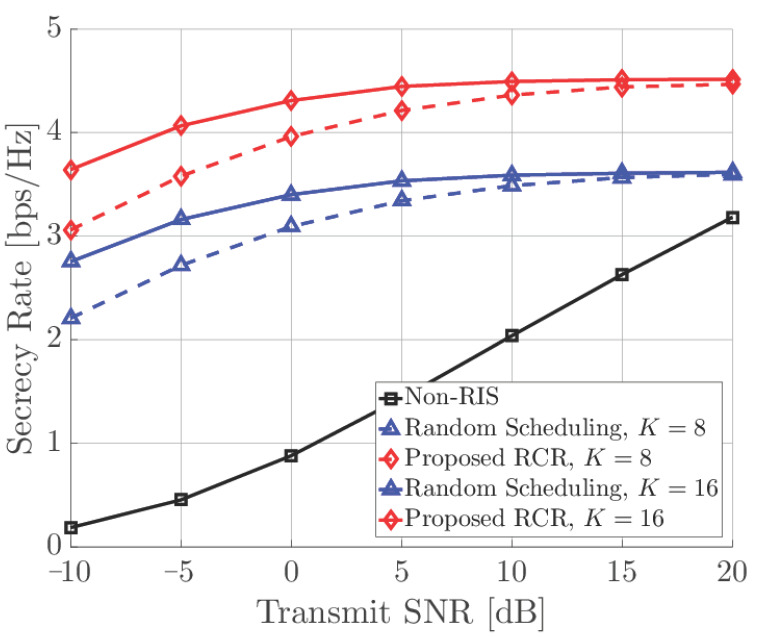
Secrecy rate performance according to transmitted SNR.

In Figure 3, the secrecy rate performance of proposed RCR technique is compared with reference techniques according to the transmit SNR (ρ), where M=4, L=16, S=1, N=10, T=10, and E=4. It is shown that performance of proposed RCR technique and that of random scheduling were saturated as transmit SNR increased. However, even the performance of the proposed RCR technique was saturated as SNR increases; it outperformed all other reference techniques; even the non-RIS case showed significant enhancement as SNR increases. Furthermore, it is shown that as the number of RISs increases, the performance of proposed RCR technique can achieve be saturated more quickly.

**Figure 4:**
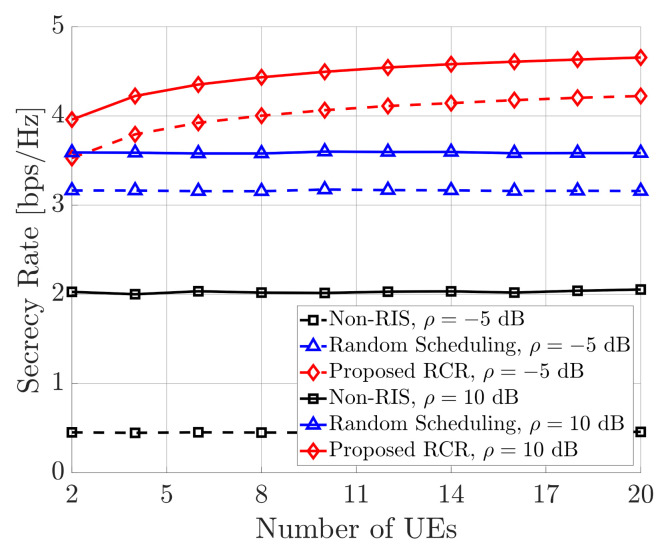
Secrecy rate performance according to number of UE.

The secrecy rate performance of proposed RCR technique according to the number of UE (*N*) is shown in Figure 4, where M=4, L=16, S=1, K=16, T=10, and E=4. Since pieces of UE are randomly scheduled in the non-RIS case and random scheduling, it is shown that their performance is not enhanced even as the number of pieces of UE increases. However, proposed RCR technique allocates the best UE for the time slot; hence, it can achieve scheduling gain from a large amount of UE. Therefore, proposed RCR technique achieved the highest performance according to number of UE.

**Figure 5:**
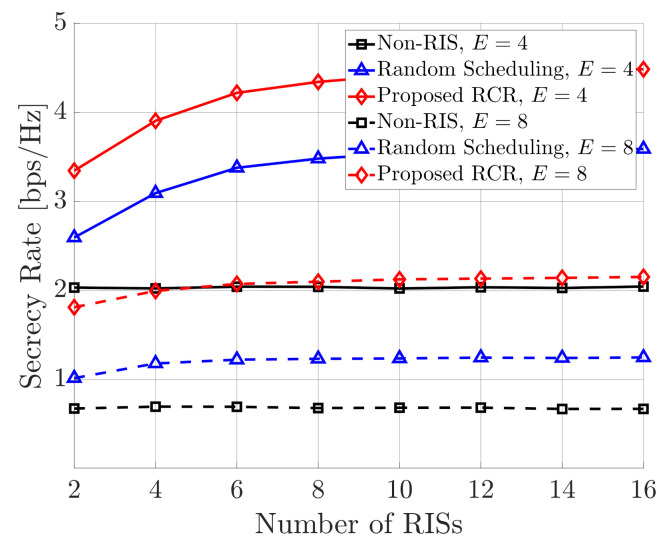
Secrecy rate performance according to number of RISs.

Figure 5 shows the secrecy rate performances of all techniques according to the number of RISs (*K*), where M=4, L=16, S=1, T=10, N=10, and ρ=10 dB. It is shown that proposed RCR technique also outperformed other reference techniques according to the number of RISs; see Figure 3 and Figure 4. Obviously, the non-RIS case is not effected by the number of RISs because it is based on the environment in which RISs do not exist.

Most importantly, the performances of the RIS-based techniques (proposed and random scheduling) do not increase linearly as the number of RISs increases, but they do get saturated. As a result, it is shown that there is no need to deploy many RISs in the network for the proposed RCR technique.

## 5. Conclusions

In this paper, we proposed a reconfigurable intelligent surface (RIS)-based channel randomization (RCR) technique for improving physical-layer security (PLS) for a time-division duplex (TDD) downlink cellular wire-tap network consisting of a single base station (BS) with multiple antennas, multiple legitimate pieces of user equipment (UE), multiple potential eavesdroppers (EVEs), and multiple RISs with multiple passive elements. In the proposed technique, each RIS pseudo-randomly generates multiple reflection matrices and utilizes pilot signal duration (PSD) in uplink and data transmission duration (DTD) in downlink for user scheduling. As a result, exactly same communication channel for the PSD will be used for the DTD so that BS can schedule the UE with information of PSD without uncertainty. In addition, the UE are scheduled to achieve the highest secrecy rate and the transmit beamforming of BS is also designed to achieve best secrecy rate for UE scheduled in a certain time slot. Through computer simulations, we validated that the proposed RCR technique achieves higher secrecy rate performance than the conventional techniques.

## Abbreviations

The following abbreviations are used in this manuscript:

AN	Artificial-noise
AP	Access point
BS	Base station
CSI	Channel state information
DTD	Data transmission duration
EVE	Eavesdropper
IoT	Internet-of-things
IRS	Intelligent reflecting surface
LOS	Line-of-sight
MISO	Multiple-input multiple-output
mmWave	Millimeter wave
NOMA	Non-orthogonal multiple access
PLS	Physical-layer security
PSD	Pilot signal duration
RCR	RIS-based Channel Randomization
RIS	Reconfigurable intelligent surface
SIMO	Single-input multiple-output
SINR	Signal to interference-plus-noise ratio
SNR	Signal-to-noise ratio
TDD	Time-division duplex
THz	Tera-hertz
UE	User equipment
ULA	Uniform linear array
